# Endothelial cell pyroptosis plays an important role in Kawasaki disease via HMGB1/RAGE/cathespin B signaling pathway and NLRP3 inflammasome activation

**DOI:** 10.1038/s41419-019-2021-3

**Published:** 2019-10-14

**Authors:** Chang Jia, Jian Zhang, Huanwen Chen, Yingzhi Zhuge, Huiqiao Chen, Fanyu Qian, Kailiang Zhou, Chao Niu, Fangyan Wang, Huixian Qiu, Zhenquan Wang, Jian Xiao, Xing Rong, Maoping Chu

**Affiliations:** 1 Pediatric Research Institute, The Second Affiliated Hospital and Yuying Children’s Hospital of Wenzhou Medical University, Wenzhou, 325027 China; 2 Children’s Heart Center, Institute of Cardiovascular Development and Translational Medicine, The Second Affiliated Hospital and Yuying Children’s Hospital of Wenzhou Medical University, Wenzhou, 325027 China; 3 University of Maryland School of Medicine, Baltimore, MD 21201 USA; 4 Department of Orthopaedics, The Second Affiliated Hospital and Yuying Children’s Hospital of Wenzhou Medical University, Wenzhou, 325027 China; 5 Department of Pathophysiology, School of Basic Medicine Science, Wenzhou Medical University, Wenzhou, Zhejiang 325000 PR China; 6 Pharmacology, School of Pharmacy, Wenzhou Medical University, Wenzhou, Zhejiang Province China

**Keywords:** Cell death, Cell death, Cardiovascular diseases, Cardiovascular diseases, Experimental models of disease

## Abstract

Kawasaki disease (KD) is the most common cause of pediatric cardiac disease in developed countries, and can lead to permanent coronary artery damage and long term sequelae such as coronary artery aneurysms. Given the prevalence and severity of KD, further research is warranted on its pathophysiology. It is known that endothelial cell damage and inflammation are two essential processes resulting in the coronary endothelial dysfunction in KD. However, detailed mechanisms are largely unknown. In this study, we investigated the role of pyroptosis in the setting of KD, and hypothesized that pyroptosis may play a central role in its pathophysiology. In vivo experiments of patients with KD demonstrated that serum levels of pyroptosis-related proteins, including ASC, caspase-1, IL-1β, IL-18, GSDMD and lactic dehydrogenase (LDH), were significantly increased in KD compared with healthy controls (HCs). Moreover, western blot analysis showed that the expression of GSDMD and mature IL-1β was notably elevated in KD sera. In vitro, exposure of human umbilical vein endothelial cells (HUVECs) to KD sera-treated THP1 cells resulted in the activation of NLRP3 inflammasome and subsequent pyroptosis induction, as evidenced by elevated expression of caspase-1, GSDMD, cleaved p30 form of GSDMD, IL-1β and IL-18, and increased LDH release and TUNEL and propidium iodide (PI)-positive cells. Furthermore, our results showed that NLRP3-dependent endothelial cell pyroptosis was activated by HMGB1/RAGE/cathepsin B signaling. These findings were also recapitulated in a mouse model of KD induced by *Candida albicans* cell wall extracts (CAWS). Together, our findings suggest that endothelial cell pyroptosis may play a significant role in coronary endothelial damage in KD, providing novel evidence that further elucidates its pathophysiology.

## Introduction

Kawasaki disease (KD) is an acute vasculitis and is the main cause of acquired heart disease in pediatric populations of developed countries^1^. Coronary artery lesions are the most severe complications in children with KD, and non-specific immune-suppressive therapy with intravenous immunoglobulin (IVIG) remains the mainstay treatment. Without early intervention, 25% of KD patients would go on to develop coronary artery aneurysm^2^, and even with prompt administration of IVIG, risk of coronary artery aneurysm remains unacceptable high (4%)^3^. Part of the difficulty of treating KD is due to its complex inflammatory pathophysiology. Previous studies reported that endothelial cell injury and inflammation are the two key pathological mechanisms for KD^4,5^. While some mechanisms for endothelial cell injury in KD, such as endothelial–mesenchymal transition (EndoMT)^2^ and endothelial cell apoptosis^6,7^, have been demonstrated, whether other mechanisms also play a role in this complex inflammatory disease remains unknown.

KD, an inflammatory disease, involves the activation of innate and adaptive immunity, which eventually causes the release of cytokine factors and establishes an inflammatory environment. Among them, the cytokine factors released by monocyte/macrophages play an important role in vascular endothelial damage during acute KD^8,9^, and THP1 cell line has been widely used as an in vitro model of human monocytes and macrophage in mechanistic studies of inflammatory disease^10^. As is known, acute inflammation in KD is accompanied by marked endothelial cell dysfunction and production of cytokines, such as interleukin (IL)-1β^11,12^, which may suggest involvement of pyroptosis in the pathophysiology of KD. Pyroptosis is a form of proinflammatory cell death that combines features of both apoptosis and necrosis, and can be mediated by inflammasome-dependent caspase-1 activation^13^, which is responsible for the cleavage and/or maturation of Gasdermin D (GSDMD), IL-1β and IL-18. Pyroptosis has been shown to play important roles in cardiovascular diseases, such as atherosclerosis^14,15^, diabetic cardiomyopathy^16^, and myocardial ischemia/reperfusion injury^17^. However, it remains unclear whether the coronary endothelial injury seen in KD is associated with pyroptosis.

Canonical pyroptosis, which is caspase-1 mediated, can be activated by seven kinds of inflammasomes, including NLRP3, NLRP1, NLRP6, NLRP9, AIM2, NLRC4, and Pyrin^13^. Among them, NLRP3 is identified as an important NOD-like receptor protein that can recognize both microbial and non-microbial danger signals and trigger a sterile inflammatory response. In a *Lactobacillus casei* (*L. casei*) cell wall extract (LCWE)-induced KD mouse model, NLRP3 inflammasome is activated in the coronary arterial endothelium and leads to a significant increase in caspase-1 activity and IL-1β production. This phenotype is rescued by *Nlrp3* gene silencing, lysosome membrane stabilizing agents, and cathepsin B silencing, indicating that LCWE induces lysosomal membrane permeabilization and subsequent lysosomal cathepsin B release, eventually leading to the activation of NLRP3 inflammasome, which then contribute to coronary arteritis in KD^18^. Pyroptosis is also known as gasdermin-mediated programmed necrosis^19^, and whether the activation of NLRP3 inflammasome also leads to endothelial necrosis is unclear in KD. Furthermore, the mechanism of lysosomal membrane rupture and cathepsin B release is also undefined.

High mobility group box 1 (HMGB1) is a highly conserved 30 kDa nuclear protein that can act as an extracellular signal after being released by immune cells or necrotic cells^20^. The released HMGB1 can mediate the proinflammatory responses of endothelial cells^21^, and induce apoptotic, autophagic or pyroptotic cell death^22–24^. In addition, HMGB1 can trigger the activation of NLRP3 and AIM2 inflammasomes after binding various DNA sequences in a TLR9 or RAGE-dependent manner^25^. Xu et al. reported that HMGB1-induced macrophage pyroptosis acts through RAGE/dynamin/cathepsin B signaling, which then triggers pyroptosome formation and caspase-1-mediated pyroptosis^21^. It has been reported that the serum contents of HMGB1 in KD patients are increased in early acute phase and declined after defervescence. Moreover, the mRNA expression of its receptor, RAGE, is remarkably upregulated in KD patients^26^. Therefore, we speculate that endothelial cell pyroptosis is induced in a NLRP3-dependent manner in KD, which is mediated by HMGB1/RAGE/cathepsin B signaling pathway.

To test our hypothesis, we first examined the levels of pyroptosis-related proteins, including ASC, caspase-1, IL-1β, IL-18, GSDMD, and lactic dehydrogenase (LDH), in the sera of KD patients as compared with healthy controls (HCs), and analyzed the expression of GSDMD and mature IL-1β by western blot analysis. Then, we confirmed pyroptosis induction in endothelial cells treated with KD-like conditions, and observed the pyroptotic changes after inhibiting NLRP3, cathepsin B, RAGE or HMGB1. To further verify the role of endothelial cell pyroptosis in KD, we detected the phenotypes and mechanisms in a murine model of KD induced by *Candida albicans* cell wall extracts (CAWS).

## Results

### Serum levels of pyroptosis-related proteins are increased in KD

**Fig. 1:**
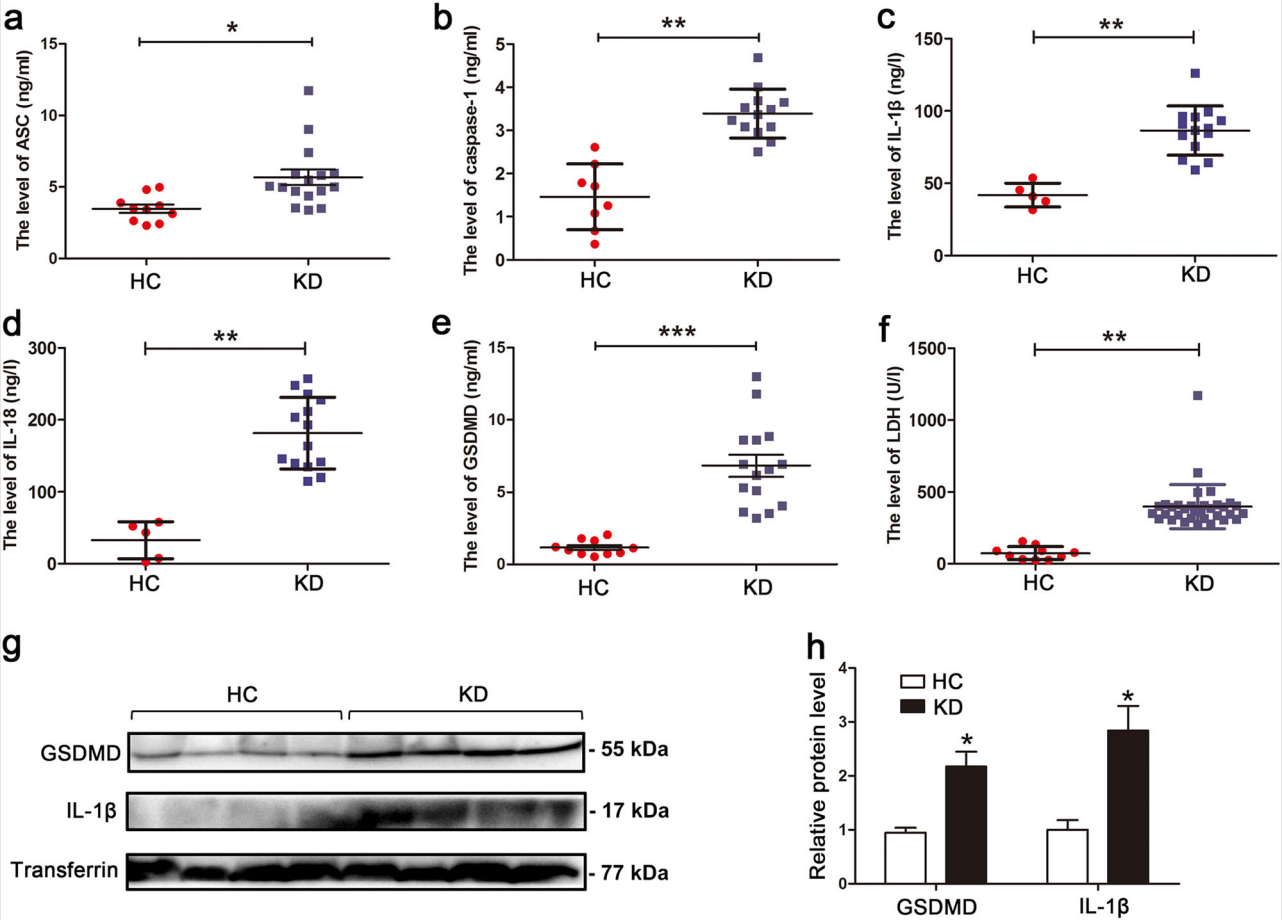
Levels of pyroptosis-related proteins were increased in sera from KD patients. **a**–**f** Enzyme-linked immunosorbent assay (ELISA) was performed to assess the levels of ASC (**a**), caspase-1 (**b**), IL-1β (**c**), IL-18 (**d**), GSDMD (**e**), and LDH (**f**) in sera from age-similar acute KD patients and healthy controls (HCs). Significance: *P < 0.05, **P < 0.01, and ***P < 0.001. **g**, **h** Expression of GSDMD and mature IL-1β was analyzed in HC (*n* = 4) and KD (*n* = 4) sera. Transferrin was applied as a loading control (**g**). The right panel is the statistical analysis of immunoblots (**h**). Data were expressed as mean ± SD. *P < 0.05

During pyroptosis, the levels of ASC, activated caspase-1, cleaved IL-1β, IL-18, and gasdermin D (GSDMD) are expected to be significantly upregulated^14^. To preliminarily determine whether pyroptosis occurs in KD, the serum levels of circulating pyroptosis-related proteins were measured and compared between KD patients and age-matched healthy controls (HCs). Results showed that the levels of total ASC, caspase-1, IL-1β, IL-18, and GSDMD were remarkably increased in sera from KD subjects compared with HC (Fig. 1a–e). Since damaged cell membrane in pyroptotic cells can lead to the release of intracellular contents^27^, including lactic dehydrogenase (LDH), LDH release assay was conducted. Here, we found that LDH levels were also significantly increased in the sera from KD patients (Fig. 1f). Western blot analysis showed that the expression of GSDMD, a key executor of pyroptosis, and mature IL-1β were also remarkably elevated in KD sera (Fig. 1g, h). These results suggested that pyroptosis may be involved in KD.

### KD sera-treated THP1 cells induce pyroptosis of endothelial cells

**Fig. 2:**
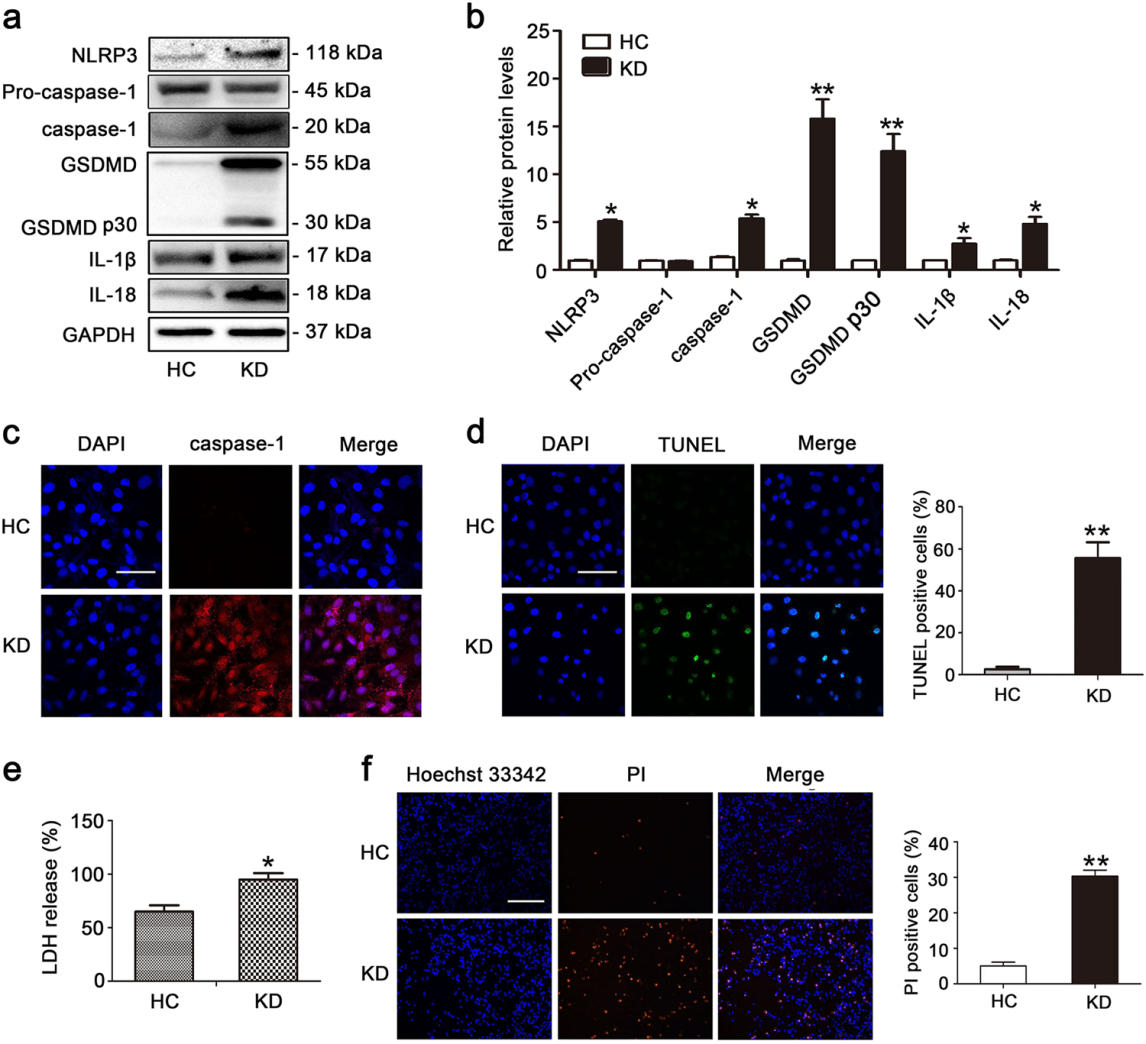
Pyroptosis occurred in human umbilical vein endothelial cells (HUVECs) after exposure to KD sera-treated THP1 cells. **a**, **b** HUVECs were co-cultured with THP1 cells that were treated with HC or KD sera (pooled from eight patients) for 24 h. Protein levels of NLRP3, caspase-1, mature IL-1β, and IL-18, and cleavage of GSDMD were examined by western blot analysis. GAPDH was used as an internal control. Data in (**b**) were shown as mean ± SD from three independent experiments involving different batches of cells but the same pooled HC or KD sera. *P < 0.05 and **P < 0.01. **c**, **d** Caspase-1 fluorescence intensity and the percentage of TUNEL-positive cells were respectively evaluated using immunofluorescent assay and TUNEL staining. The nuclei were stained blue using DAPI. Magnification: × 200. Scale bar = 100 μm. **P < 0.01. **e** LDH release was determined in HUVECs after HC or KD sera treatment (*n* = 5). *P < 0.05. **f** Percentage of PI-positive cells was detected in HUVECs using Hoechst33342/PI staining. Magnification: ×100. Scale bar = 500 μm. Significance: **P < 0.01

Next, we conducted in vitro experiments to further investigate the role of pyroptosis in KD. In KD, endothelial cells are exposed to inflammatory conditions. Thus, to re-create the inflammatory environment surrounding endothelial cells in KD, we utilized KD sera-treated THP1 cells and co-cultivated them with human umbilical vein endothelial cells (HUVECs) to create an in vitro coculture experimental system. More specifically, these two cell types were separated by a transwell chamber, with the HUVECs were cultured in the lower chamber, and the THP1 cells were placed in the upper chamber that permits diffusion of soluble molecules. To characterize whether pyroptosis was induced in the endothelial cells (ECs) after co-culturing them with KD sera-treated THP1 cells (referred to as “KD-treated ECs” from now on), the expression of pyroptosis-related proteins was assessed by western blot analysis. As shown in Fig. 2a, b, the expression levels of NLRP3, caspase-1, GSDMD, cleaved p30 form of GSDMD, IL-1β and IL-18 were notably increased in KD-treated ECs. To further dissect EC pyroptosis, the immunofluorescence staining of caspase-1 and TUNEL, LDH release and PI staining were respectively evaluated^11^. Results showed that caspase-1 fluorescence intensity, TUNEL-positive cells, release of LDH, and PI-positive cells were remarkably elevated by KD conditions (Fig. 2c–f). To further substantiate that pyroptosis was implicated in the above phenotypes, GSDMD-derived pyroptosis inhibitors, necrosulfonamide and Ac-FLTD-CMK, were utilized to observe EC viability. As shown in Figs. S1 and S2, the addition of these pyroptosis inhibitors significantly decreased LDH release, increased cell viability, alleviated cell death and declined IL-1β release. Together, these results suggested that pyroptosis-related cell death occurred in KD-treated ECs in our in vitro model of KD.

### NLRP3 inflammasome activation triggers downstream activation of pyroptosis in KD-treated ECs

**Fig. 3:**
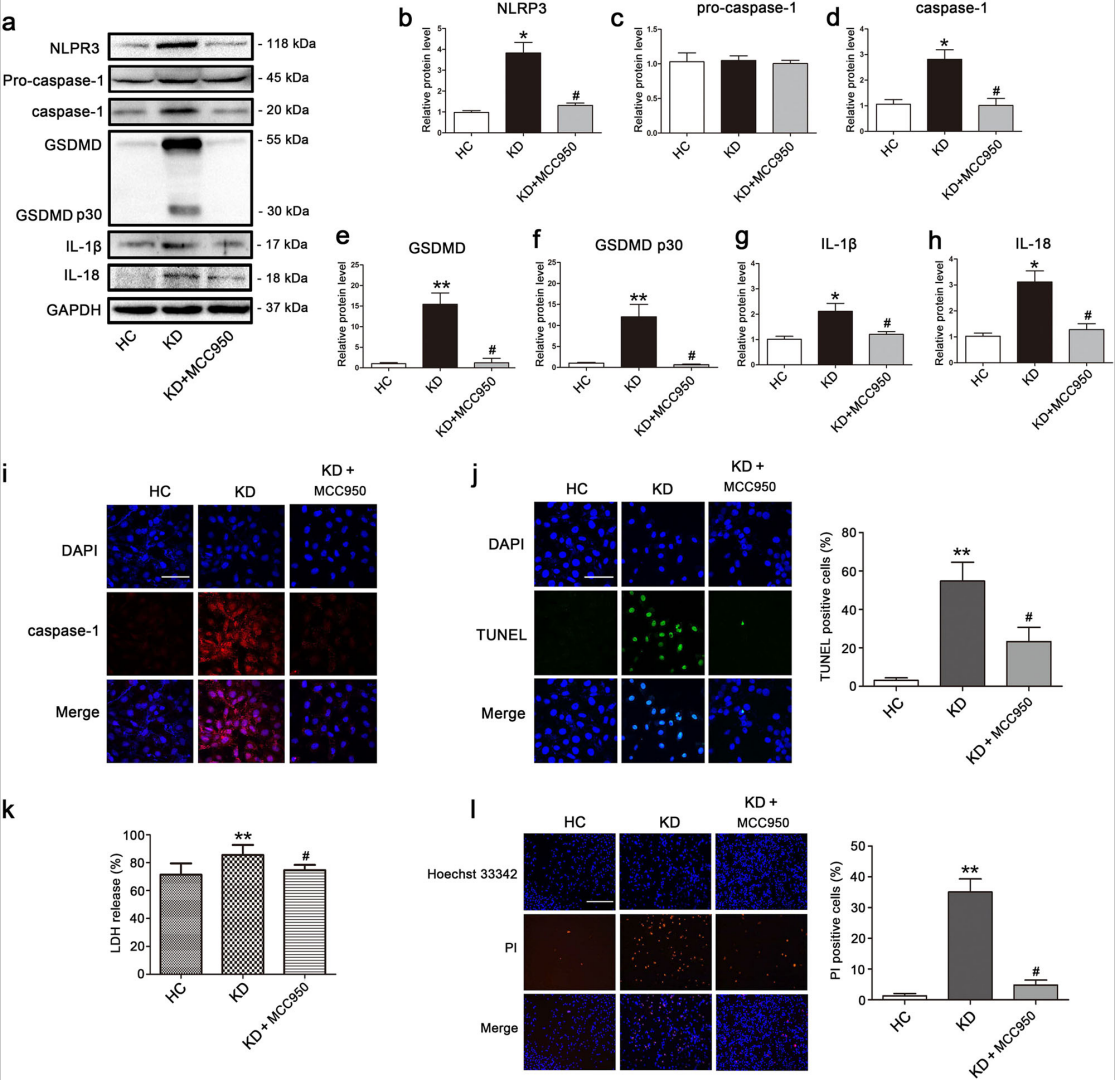
NLRP3 inhibitor repressed KD-treated EC pyroptosis. HUVECs were pretreated with NLRP3 inhibitor (MCC950, 10 μM) for 30 min, and then ECs were co-incubated with pooled sera-treated THP1 for 24 h. **a** Effect of MCC950 pretreatment on the protein expression of pyroptosis were analyzed by western blot analysis. GAPDH was used as an internal control. **b**–**h** Quantitative analysis of pyroptosis-related protein expression was conducted. Data were presented as mean ± SD (*n* = 3). **i**–**k** Caspase-1 expression, DNA fragmentation and LDH release were determined using immunofluorescent assay, TUNEL staining and LDH activity assay kits, respectively. Magnification: ×200, Scale bar = 100 μm. **l** Double staining of Hoechst 33342 (blue) and PI (red) was conducted in the treated endothelial cells (Left: the representative photographs, Right: the quantification evaluation of PI-positive cells). Magnification: ×100, Scale bar = 500 μm. Significance: *P < 0.05 and **P < 0.01 indicates significant difference between KD group and HC group, and ^#^*P* < 0.05 indicates significant difference between KD groups with and without MCC950 addition

NLRP3 inflammasome is known to recruit and activate caspase-1 through ASC, and activated caspase-1 can then cleave GSDMD, IL-1β, and IL-18 to their mature forms and subsequently trigger pyroptotic cell death^28^. Given our results confirming the concurrent activation of NLRP3, caspase-1, IL-1β, IL-18, and GSDMD, we hypothesized that NLRP3 inflammasome may be triggered upstream of pyroptosis activation. To substantiate whether caspase-1 activation and subsequent pyroptosis were mediated by upstream NLRP3 inflammasome activation, we performed NLRP3 inhibitory experiments with the NLRP3 inhibitor MCC950. Results showed that MCC950 addition notably decreased the protein level of NLRP3, activated caspase-1, GSDMD, and cleaved p30 form of GSDMD, and inhibited the maturation of IL-1β and IL-18 (Fig. 3a–h). Moreover, caspase-1 fluorescence intensity and the percentage of TUNEL-positive cells were obviously reduced by treatment with MCC950 in KD-treated ECs (Fig. 3i, j). What’s more, LDH release and PI-positive cells were reduced as well (Fig. 3k, l), indicating that the cell membrane rupture and cell lysis were reversed by MCC950. Together, these data provided strong evidence that EC pyroptosis in KD conditions was downstream effect of NLRP3 inflammasome activation.

### Pyroptosis of KD-treated ECs is dependent on cathepsin B activation

**Fig. 4:**
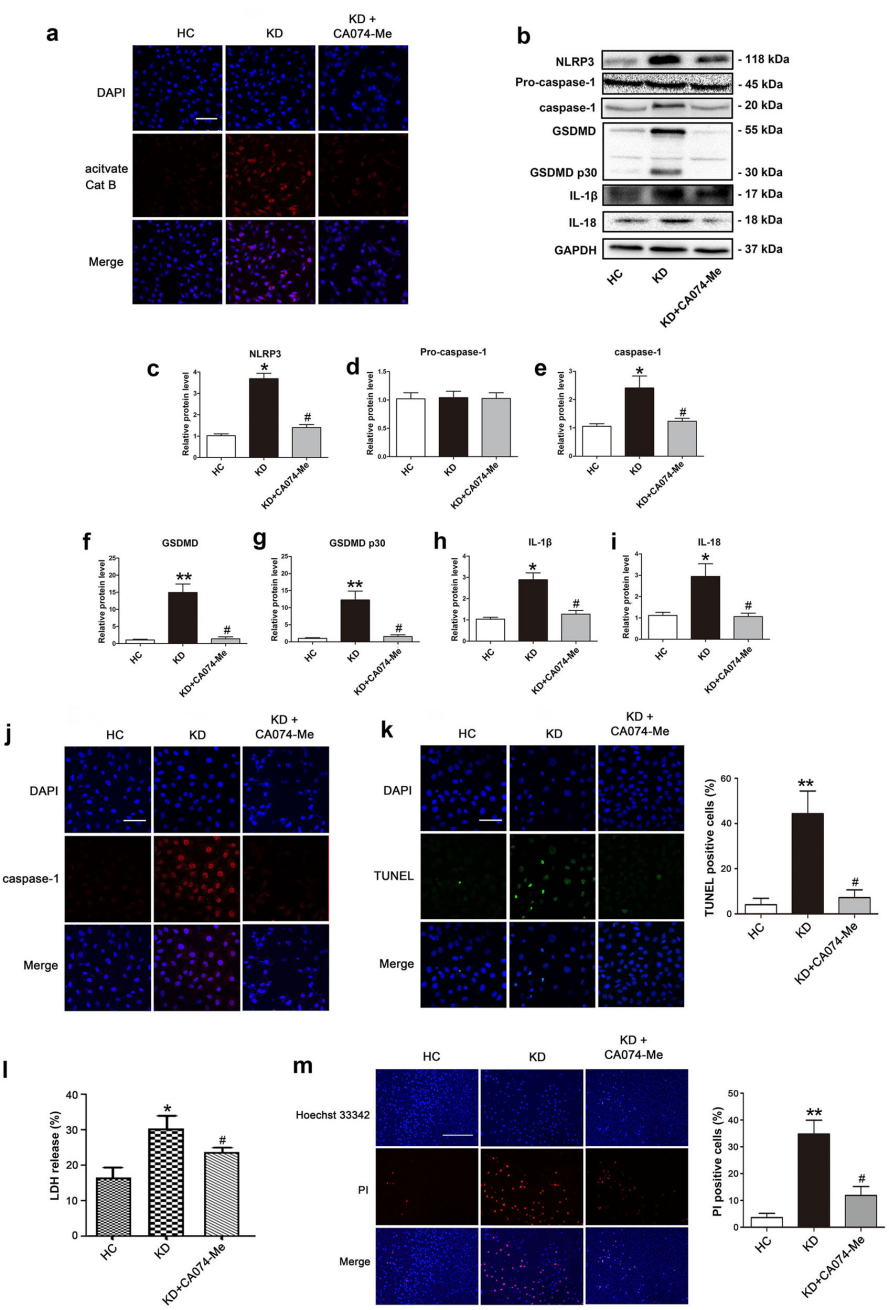
Inhibition of cathepsin B alleviated KD-treated EC pyroptosis. HUVECs were pretreated with a cathepsin B inhibitor (CA074-Me, 25 μM) for 30 min, and then ECs were co-cultured with pooled sera-treated THP1 for 24 h. **a** Endothelial cells were stained with Magic Red Cathepsin B detection reagent to examine the activation of cathepsin B. Magnification: ×200, Scale bar = 100 μm. **b** Effects of CA074-Me treatment on the expression of pyroptosis-associated proteins were assessed in the treated endothelial cells. **c**–**i** Quantitative analysis of pyroptosis-related protein expression, and GAPDH was used as an internal control. Data were exhibited as mean ± SD (*n* = 3). **j**–**m** Caspase-1 expression, TUNEL staining, LDH release, and Hoechst 33342/PI double staining were conducted as described in Fig. 2c–f. Magnification: ×200, Scale bar = 100 μm for (**j**) and (**k**). Magnification: ×100, Scale bar = 500 μm for (**m**). Significance: *P < 0.05, and **P < 0.01 indicate significant difference between KD group and HC group. ^#^*P* < 0.05 indicates significant difference between KD groups with and without CA074-Me addition

Lysosome rupture, and cathepsin B activation and release from lysosomes are important mechanisms for the activation of NLRP3 inflammsome^29^. Previous study has reported that endothelial NLRP3 inflammasome activation is related to lysosomal destabilization and consequent release of lysosomal cathepsin B in an animal model of KD (coronary arteritis induced by LCWE)^18^. To address whether NLRP3 inflammasome-mediated EC pyroptosis found in our model is associated with cathepsin B activation in KD, cathepsin B activity in the cytoplasm was examined using Magic Red Cathepsin B detection reagent. KD-treated ECs exhibited an obvious cytosolic pattern of activated cathepsin B staining compared with cells treated with control while the addition of cathepsin B specific inhibitor CA074-Me significantly inhibited cathepsin B activity (Fig. 4a). Moreover, when ECs were pretreated with CA074-Me, the protein levels of pyroptosis-related proteins, including NLRP3, caspase-1, GSDMD, cleaved p30 form of GSDMD, mature IL-1β and IL-18, were remarkably decreased in KD sera-treated cells (Fig. 4b–i). Furthermore, caspase-1 fluorescence intensity and DNA fragmentation detected by TUNEL were significantly attenuated after pretreatment with CA074-Me (Fig. 4j, k). In addition, the level of LDH release and cell membrane damage indicated by PI staining were also decreased by CA074-Me (Fig. 4l, m). These results confirmed a key role of cathepsin B for regulating pyroptosis in KD.

### Pyroptosis of KD-treated ECs is mediated by HMGB1/RAGE signaling

**Fig. 5:**
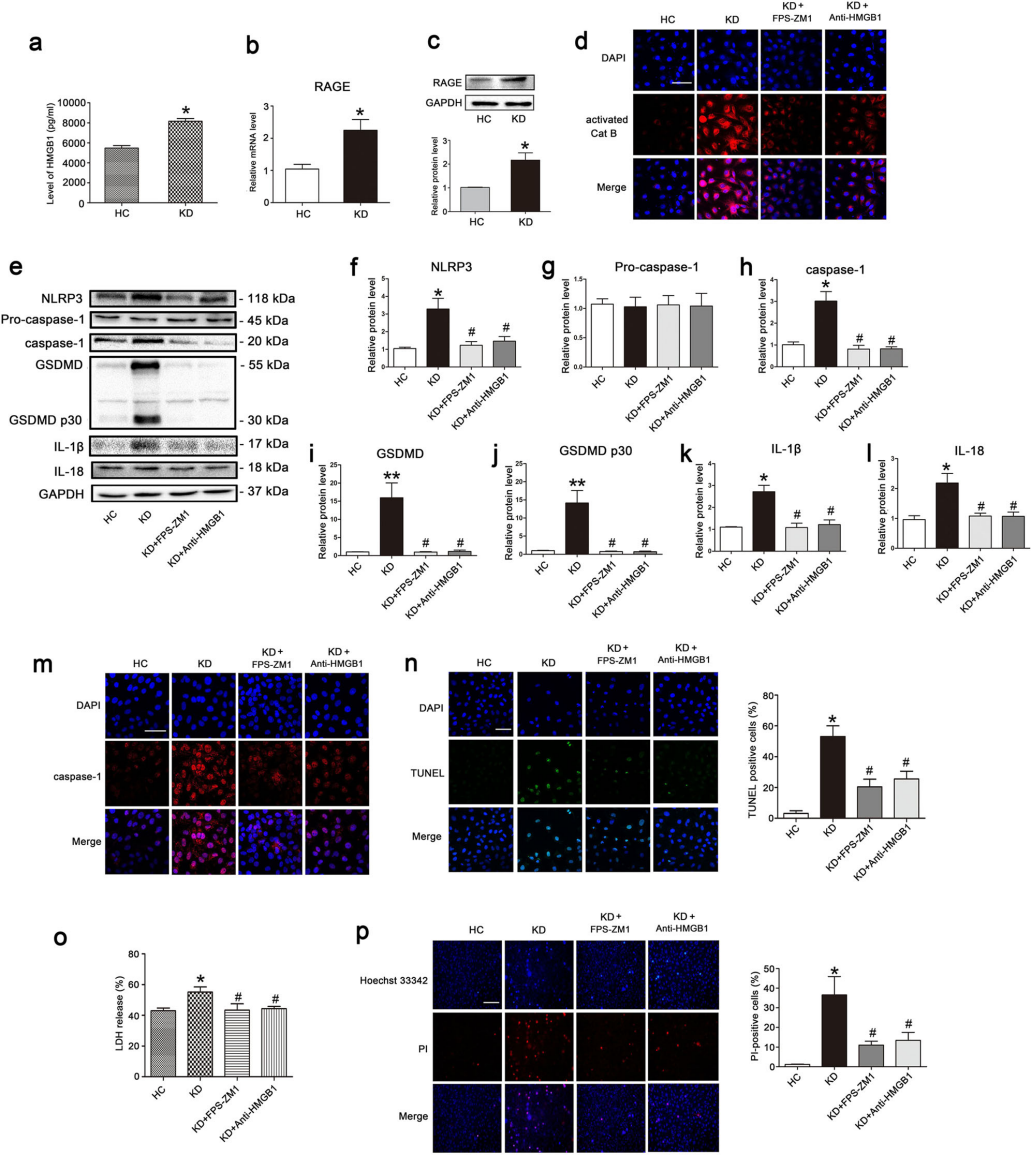
KD-treated EC pyroptosis was dependent on HMGB1/RAGE signaling. **a** HMGB1 contents were evaluated in the supernatant medium of pooled sera-treated THP1 cells by ELISA analysis. **b**, **c** The mRNA and protein expressions of RAGE were determined in ECs. **d** Effects of a RAGE inhibitor (FPS-ZM1) or the anti-HMGB1 antibody on the activation of cathepsin B were assessed. **e** Protein expression levels of NLRP3, caspase-1, GSDMD, IL-1β, and IL-18 in the treated endothelial cells were determined. **f**–**l** Expression of pyroptosis-related proteins was quantitatively analyzed, and GAPDH was used as an internal loading control. Data were exhibited as mean ± SD (*n* = 3). **m**–**p** Caspase-1 expression, TUNEL staining, LDH release, and Hoechst 33342/PI double staining were performed according to the above descriptions. Magnification: ×200, Scale bar = 100 μm for (**m**) and (**n**). Magnification: ×100, Scale bar = 200 μm for (**p**). Significance: *P < 0.05, **P < 0.01 vs. the HC group. ^#^*P* < 0.05 vs. the KD group

It is well known that serum levels of HMGB1 are increased in KD during the early acute phase and gradually declines after defervescence. In particular, the expression of one of HMGB1 receptors, RAGE, is upregulated in KD patients^26^, suggesting that HMGB1-RAGE signaling may play an important role in the pathophysiology of KD. Previous studies demonstrated that HMGB1-induced macrophage pyroptosis is mediated by RAGE-dependent signaling, which can initiate HMGB1 endocytosis and subsequent cathepsin B release from damaged lysosomes^24^. To decipher whether our results of pyroptosis induction in KD-treated ECs is related to HMGB1-RAGE signaling, HMGB1 levels in the supernatant medium of KD sera-treated THP1 cells, and RAGE expression in ECs were determined. Results showed that HMGB1 contents were significantly increased in the supernatant of THP1 cells after treatment with KD sera (Fig. 5a), and the mRNA level and protein expression of RAGE was notably elevated in ECs after treatment with KD conditions (Fig. 5b, c). To determine whether HMGB1/RAGE signaling is responsible for pyroptosis in KD-treated ECs, we pretreated ECs with a RAGE-specific inhibitor (FPS-ZM1) or an anti-HMGB1 neutralizing antibody. Then, we measured cathepsin B activity, along with the expression levels of pyroptosis-related proteins, including NLRP3, caspase-1, GSDMD, cleaved p30 form of GSDMD, mature IL-1β and IL-18. Results showed that these parameters were remarkably reduced in KD-treated ECs (Fig. 5d–l). Moreover, the activated caspase-1 fluorescence intensity, DNA fragmentation, LDH release, and membrane rupture were also remarkably alleviated after inhibition of RAGE by FPS-ZM1 or neutralization of HMGB1 by the anti-HMGB1 antibody (Fig. 5m–p). Together, these results demonstrated EC pyroptosis in KD was realized by HMGB1/RAGE signaling, which resulted in subsequent cathepsin B activation and NLRP3-mediated EC pyroptosis.

### EC pyroptosis is NLRP3-dependent in a KD mouse model

**Fig. 6:**
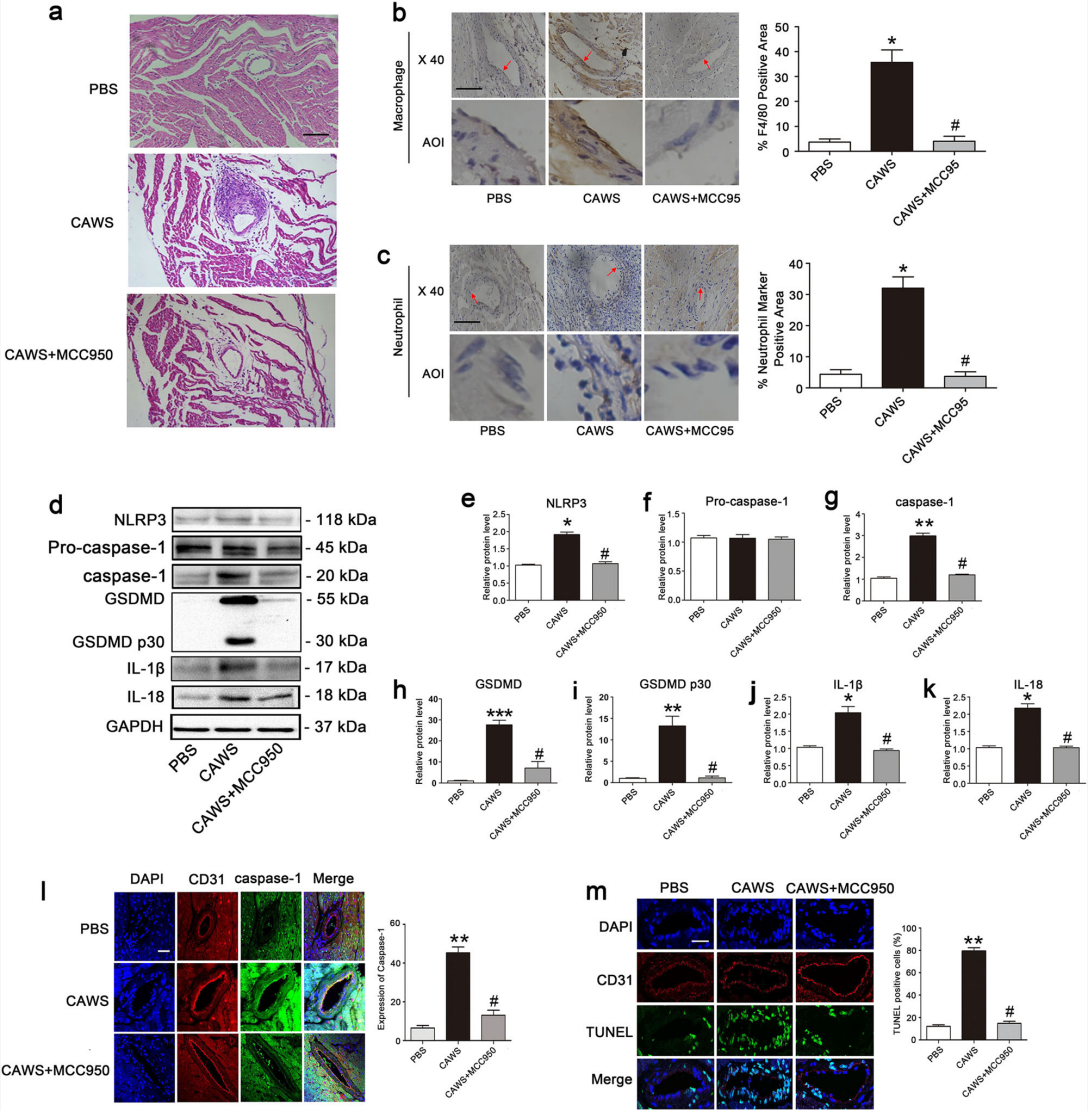
NLRP3-mediated endothelial cell pyroptosis in a KD mouse model. Mice were continuously injected i.p. with 4 mg CAWS for 4 days to induce coronary arteritis. Twenty-eight days later, mice were sacrificed and the heart tissues were harvested for follow-up examinations. **a** Representative images from H&E staining at 28 day post-CAWS injection. Scale bar = 100 μm. **b** Expression of macrophage marker F4/80 was determined using IHC staining in the endothelium of coronary arteries. The histogram showed the area percentage of the endothelium positive for F4/80 in coronary arteries. Enlarged images of area of interesting (AOI) were indicated with a red arrow. Scale bar = 100 μm. **c** The expression of neutrophil marker was examined in the endothelium of coronary arteries. The histogram exhibited the area percentage of the endothelium positive for neutrophil marker in coronary arteries. Scale bar = 100 μm. **d** Expression of pyroptosis-related proteins was examined by western blot analysis. **e**–**k** Quantitative analysis of pyroptosis-related protein expression was conducted. Data were expressed as mean ± SD (*n* = 3). **l** Comparison of expression and subcellular distribution of caspase-1 by double staining of caspase-1 (green) and CD31 (red) in coronary artery of control mice, CAWS mice, and CAWS mice pretreatment with MCC950. The presence of caspase-1 in endothelial cells was indicated by the co-localization of caspase-1 and CD31 (an endothelial marker). Magnification: ×200. Scale bar = 50 μm. **m** Identification of DNA fragmentation by co-localized staining of TUNEL (green) and CD31 (red). The nuclei were stained blue with DAPI. Magnification: ×200. Scale bar = 50 μm. Significance: *P < 0.05, **P < 0.01 vs. the control group. ^#^*P* < 0.05 vs. the CAWS group

To further explore the role of pyroptosis in a mouse model of KD, *Candida albicans* cell wall extracts (CAWS) were used to induce coronary arteritis as described before^5^. Consistent with previous studies, CAWS treatment obviously induced coronary arteritis. However, the addition of NLRP3 inhibitor MCC950 significantly attenuated coronary artery inflammation (Fig. 6a). Further study found the involved inflammatory cells included macrophages (Fig. 6b) and neutrophils (Fig. 6c). To investigate whether the mitigated coronary arteritis was associated with pyroptosis, the expressions of pyroptosis-related proteins were determined. As anticipated, the protein levels of NLRP3, activated caspase-1, GSDMD, cleaved p30 form of GSDMD, IL-1β, and IL-18 were significantly upregulated in the KD mouse model, and MCC950 treatment significantly decreased the expression of these proteins (Fig. 6d–k). To further demonstrate that pyroptosis occurred in coronary endothelium, CD31/caspase-1 double staining and CD31/TUNEL double staining were performed. As shown in Fig. 6l, m, the expression level of activated caspase-1 and the percentage of TUNEL-positive cells were markedly increased in the coronary endothelium of the KD mouse model, and the supplementation of MCC950 significantly decreased active caspase-1 level and TUNEL-positive cells. Collectively, these results indicated that coronary EC pyroptosis played a role in the KD mouse model, and that pyroptotic cell death in ECs was mediated by the NLRP3 inflammasome.

### NLRP3 inflammasome-mediated EC pyroptosis is realized through HMGB1/RAGE/cathepsin B axis in the KD mouse model

**Fig. 7:**
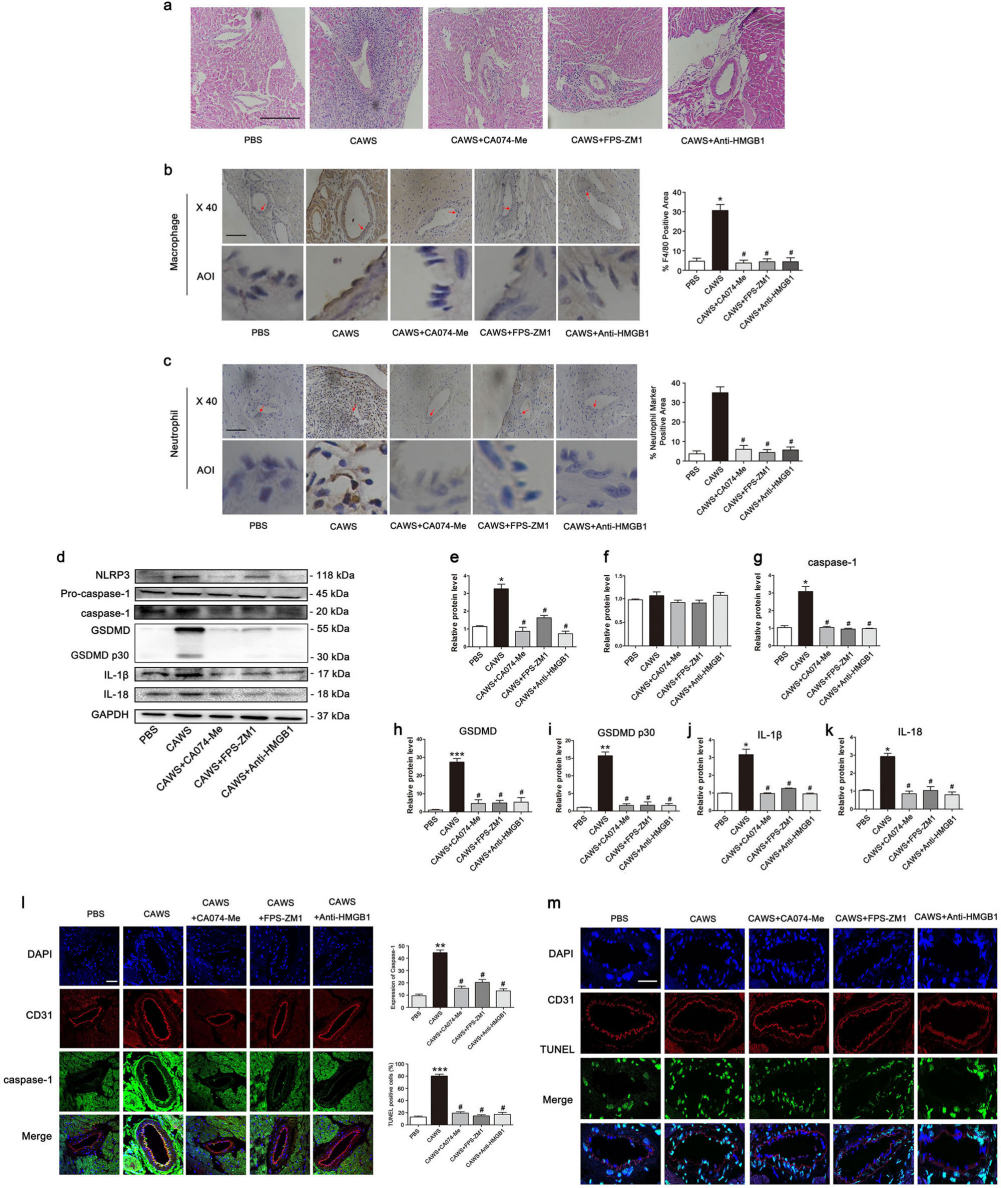
HMGB1/RAGE/cathepsin B signaling was implicated in the endothelial cell pyroptosis in the KD mouse model. Mice were pretreated with a cathepsin B inhibitor (CA074-Me), a RAGE-specific inhibitor (FPS-ZM1), and the anti-HMGB1 antibody 60 min before CAWS injection. **a** Heart tissues were analyzed for inflammatory infiltration by H&E staining. Magnification: ×200. Scale bar = 200 μm. **b** Expression of macrophage marker F4/80 was determined using IHC staining in the endothelium of coronary arteries. The histogram showed the area percentage of the endothelium positive for F4/80 in coronary arteries. Enlarged images of area of interesting (AOI) were indicated with a red arrow. Scale bar = 100 μm. **c** The expression of neutrophil marker was examined in the endothelium of coronary arteries. The histogram exhibited the area percentage of the endothelium positive for neutrophil marker in coronary arteries. Scale bar = 100 μm. **d** Western blot analysis was used to determine the expression of pyroptosis-related proteins. **e**–**k** Quantitative analysis was performed to detect pyroptosis-related protein expression. Data were shown as mean ± SD (*n* = 3). **l** Caspase-1 expression was analyzed in coronary endothelial cells using caspase-1/CD31 double staining. Magnification: × 200. Scale bar = 50 μm. **m** DNA fragmentation in endothelial cells was identified by co-localization observation of TUNEL and CD31. The nuclei were stained blue with DAPI. Magnification: ×200. Scale bar = 50 μm. Significance: *P < 0.05, **P < 0.01, ***P < 0.001 vs. the control group. ^#^*P* < 0.05 vs. the CAWS group

To confirm whether our in vitro observation of HMGB1/RAGE/cathepsin B signaling-mediated EC pyroptosis also occurs in vivo, KD mice were intraperitoneally injected with a cathepsin B inhibitor (CA074-Me), a RAGE-specific inhibitor (FPS-ZM1), or the neutralizing antibody against HMGB1 60 min before CAWS treatment. Results showed that the addition of these inhibitors significantly alleviated coronary inflammation (Fig. 7a), and the adhesion of macrophages and neutrophils (Fig. 7b, c). Moreover, the expression of pyroptosis-related proteins was also obviously downregulated after treatment with these inhibitors in the KD mouse model (Fig. 7d–k). Furthermore, pyroptotic cell death in the coronary endothelium was remarkably weakened as evidenced by the decreased fluorescence intensity of activated caspase-1 and reduced percentage of TUNEL-positive cells (Fig. 7l, m). Taken together, these results provided firm evidence that HMGB1/RAGE/cathepsin B signaling played an important role in activating EC pyroptosis in KD.

## Discussion

**Fig. 8:**
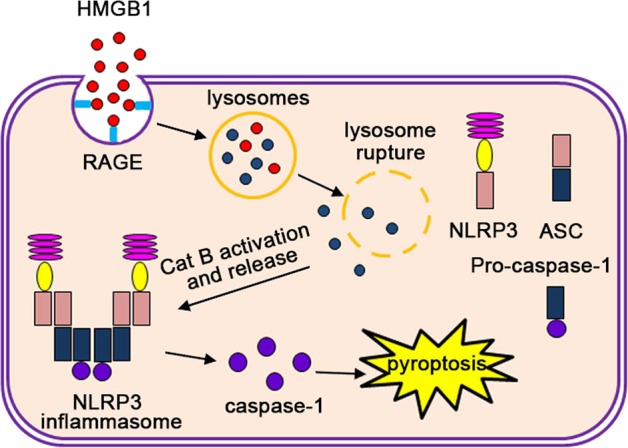
Schematic model for endothelial cell pyroptosis in Kawasaki disease. High level of serum HMGB1 interacts with its receptor RAGE, enters lysosomes, and induces Cat B (cathepsin B) activation and release from the ruptured lysosomes. This is followed by NLRP3 inflammasome activation and subsequent induction of caspase-1-dependent pyroptosis

In the present study, we demonstrated that EC pyroptosis is a crucial pathophysiological event in KD, and that activation of pyroptosis is triggered by high levels of HMGB1, leading to elevated expression of RAGE and cathepsin B activity, which results in NLRP3 inflammasome-dependent caspase-1-mediated pyroptotic cell death in the ECs (Fig. 8). This previously unappreciated cellular mechanism sheds new light on the pathophysiology of KD, and may open new avenues for the development of techniques to diagnose, evaluate, and treat this potentially devastating disease.

KD is an acute systemic vasculitis that involves inflammatory environment and endothelial damage. Innate and adaptive immune cells are activated, and inflammatory cytokines are released by a variety of cells, including ECs. These events can be attributed to activation of pyroptosis, and our results showed that pyroptosis-related parameters were significantly upregulated in the sera from KD patients. To prove upregulation of pyroptosis-related proteins was associated with vascular endothelial damage, monocyte/macrophages (THP1 cell lines) that are essential for the induction of an inflammatory condition in KD were utilized to simulate the effects of in vivo inflammatory environment on ECs. Results showed that the expression of pyroptosis-related proteins were upregulated in our in vitro model of KD-treated ECs. Attention should be paid to the upregulation of GSDMD in addition to its cleaved form. Previous studies reported that the transcriptional expression of GSDMD is under the control of nuclear factor κB (NF-κB)^30^. The elevated expression of GSDMD revealed that activation of NF-κB signal in KD, in line with previous reports^31^. To further demonstrate that EC death was associated with pyroptosis, GSDMD-derived pyroptosis inhibitors were added. Addition of these pyroptosis notably inhibited EC death, the expression of pyroptosis-related parameters, and IL-1β release (Figs. S1 and S2), indicating that pyroptosis occurred in KD-treated ECs. Pyroptotic cell death may be triggered by the activation of canonical caspase-1 and non-canonical caspase-4/511 (human caspase-4/5 and murine caspase-11). In brief, the canonical signaling pathway involves activation of NLRP1, NLRP6, NLRP9, AIM2, NLRC4, Pyrin, and NLRP3. These molecules respond to a variety of signals, such as toxins^32^, microbes^33,34^, double strand RNA and DNA sequences^35,36^, RhoA GTPase^37^, and a variety of other agents. Our data showed that the expression levels of NLRP3 were increased in KD, and inhibition of NLRP3 remarkably decreased the level of pyroptosis-related parameters, and alleviated inflammation in the coronary arteries. These results suggest that EC pyroptosis seen in KD is most likely associated with the activation of NLRP3 inflammasome.

NLRP3 can be activated by crystal phagocytosis, ROS (reactive oxygen species) production, potassium or calcium ion movement, or lysosome disruption and cathepsin B release^38^. Previous studies demonstrated that the NLRP3 activation seen in LCWE-induced KD animal models cannot be ameliorated by potassium channel blockade or ROS scavengers. However, lysosome membrane stabilizing agents and cathepsin B inhibitors can abolish the induced activation of NLRP3 inflammasome in the coronary artery endothelium, indicating NLRP3 activation was mediated by lysosome membrane rupture and the released cathepsin B in KD^18^. Activated cathepsin B can regulate the activation of NLRP3 inflammasome directly through interaction with the LRR domain of NLRP3^29,39^, or indirectly via calcium signaling^40^ or oxidative stress^41^. Our study showed that the activity of cathepsin B was increased in KD-treated ECs, and the addition of cathepsin B inhibitor significantly downregulate the expression of pyroptosis-related proteins, including NLRP3, activated caspase-1, GSDMD, cleaved p30 form of GSDMD, IL-1β, and IL-18. In addition, inhibition of cathepsin B (CA074-Me) also alleviated EC membrane rupture, and coronary artery inflammation was also rescued in our animal model of KD, together suggesting EC pyroptosis in KD is mediated by cathepsin B activation, which subsequently activated the NLRP3 inflammasome.

Cathepsin B, a member of the cathepsin protease family, resides mainly in lysosomes, and is released upon lysosomal rupture^42^. Lysosomal membranes can be permeabilized by a variety of means, including lysosomotropic detergents, viral proteins, toxin, ROS and so on^43^. Previous studies have reported that HMGB1 can permeabilize the phospholipid bilayer in the acidic environment of lysosomes, and trigger the leakage of LPS into the cytosol^44^. Xu et al. also demonstrated that HMGB1 endocytosis leads to lysosome rupture, cathepsin B release, and subsequent caspase-1-mediated pyroptotic cell death in macrophages^24^. Our data and previous studies have demonstrated that the serum level of HMGB1 is significantly increased in KD (Fig. S5b)^26^. Our results also showed that the level of HMGB1 in the supernatant medium and cultured THP1 cells was elevated (Fig. 5a and Fig. S5a). HMGB1 is known to be able to signal through the RAGE receptor, which are present in ECs. In KD, it has been reported that RAGE mRNA expression is increased^26^, and our data also showed that the mRNA and protein levels of RAGE in ECs were upregulated in KD conditions, which might be induced by elevated HMGB1 since HMGB1 internalization is RAGE-dependent, and RAGE deficiency can prevent HMGB1 endocytosis, and subsequent pyrotposis^21^. Furthermore, we found that either anti-HMGB1 antibody or RAGE inhibition in KD significantly ameliorated the inflammation in coronary arteries, decreased the expression of pyroptosis-related proteins, and alleviated EC pyroptosis. Together, these results suggest that EC pyroptosis in KD is mediated by HMGB1/RAGE signaling, and is an important cause of coronary artery injury.

Collectively, our data provides new evidence that pyroptosis of ECs is a crucial player in the pathophysiology of KD. Our results demonstrate that HMGB1 released by immune cells triggers the HMGB1/RAGE signaling pathway in ECs, which then induces cathepsin B activation, subsequently activating canonical pyroptosis via the NLRP3 inflammasome. Given that KD is diagnosed using clinical criteria, and is thus prone to subjectivity, biomarkers that are specific to KD pathophysiology would be extremely valuable to optimize the management of KD patients. Our new findings suggest that circulating HMGB1 may be a sensitive predictor for EC damage in KD. Furthermore, mechanistic insights into KD outlined in this study also open new possibilities for therapeutic targets that act on inhibiting pyroptosis activation. These exciting new opportunities await further research and subsequent clinical translation. However, there are some limitations in our current study. Whether other factors or non-canonical pyroptosis signals are also involved in EC damage of KD remains uncertain, and requires further investigation. In addition, this study cannot exclude that other forms of cell death, such as apoptosis and necroptosis, also play an important role in pathophysiology of KD. Nevertheless, our current study presents novel evidence that firmly demonstrates the involvement of EC pyroptosis in KD.

## Materials and methods

### Patients’ blood samples and ethical considerations

Blood samples and clinical information were acquired from patients with KD and age-matched healthy controls at the Second Affiliated Hospital and Yuying Children’s Hospital of Wenzhou Medical University between August 2017 and August 2019. The average age of KD patients and HCs was, respectively, 24.6 ± 10.23 and 22.5 ± 13.4 months. Healthy subjects were all children who received regular health checks and had no infections. Children that define KD met the criteria developed in 2017 by the American Heart Association^3^. All KD patients were scheduled to receive both aspirin (30–50 mg/kg/day) and intravenous immunoglobin (IVIG, 2 g/kg). All these patients were IVIG-sensitive, and had no coronary aneurysm and additional comorbid medical conditions. Two microliters of serial blood specimens were collected from every KD patient in the acute phase (before IVIG therapy on days 3–7). All sera samples were stored at −80 °C within 4 h following collection until later use. All participants gave written consent for the use of their clinical information and blood samples for academic research. This research was approved by the ethics committee of Wenzhou Medical University, and conducted in accordance with the Helsinki Declaration.

### Enzyme-linked immunosorbent assay (ELISA)

Serum concentrations of ASC, caspase-1, IL-1β, IL-18, and GSDMD, and HMGB1 levels in the supernatant medium of THP1 cells were determined by the corresponding ELISA kits according to the manufacturer’s instructions. ELISA kits for human ASC, human IL-1β, human IL-18, and human GSDMD were acquired from ABclonal Biotechnology Co., Ltd. (Boston, MA, USA), and ELISA kits for human caspase-1 and human HMGB1 were purchased from Westang Bio-Technology Co., Ltd. (Shanghai, China). For examining HMGB1 levels in the THP1 cell supernatants, experiments were conducted as follows. Specifically, the HC and KD sera were removed and THP1 cells were washed with PBS after treatment with sera for 24 h. Next, the THP1 cells continued to be incubated for the subsequent 24 h. After that, HMGB1 levels were determined in the supernatants.

### Cell culture and treatments

Human umbilical vein endothelial cells (HUVECs) and the human monocytic leukemia cell line, THP1, were purchased from American Type Culture Collection (ATCC, Manassas, VA, USA). Authentication of these cell lines was performed by the Genetic Testing Biotechnology Corporation (Suzhou, China) and the KeyCen BioTech (Nanjing, China) by short tandem repeat (STR) markers, and no mycoplasma contamination was detected. HUVECs were cultured in high glucose Dulbecco’s modified Eagle’s medium (DMEM) supplemented with 10% fetal bovine serum (FBS), and 1% (v/v) pencillin/streptomycin at 37 °C with 5% CO_2_/95% air. HUVECs were plated in 6-, 24-well plates (Becton Dickinson Labware, Franklin Lakes, NJ) for different experiments, including LDH release, caspase-1 staining, TUNEL staining, cathepsin B staining and Hoechst 33342/PI staining. THP1 cells were maintained in 75-cm^2^ plastic tissue culture flasks in RPMI-1640 medium with 10% FBS. In the coculture system, HUVECs were cultured in the lower chamber, and the THP1 cells were placed in the upper chamber that permits diffusion of soluble molecules. Then KD or HC sera were added to the upper chamber and treated THP1 cells for 24 h. At the same time, the HUVECs in the lower chamber would be affected by the diffusing molecules. All cultures were grown in DMEM medium. If necessary, the HUVECs would be pretreated with inhibitors for 30 min, and then co-cultured with KD serum- or HC serum-treated THP1 cells for 24 h.

### Western blot analysis

Total proteins were extracted from heart tissues or endothelial cells using protein extraction reagents. Protein concentrations were quantified, and equal amounts of proteins (60 μg) were separated by 12% SDS-PAGE gel and electro-transferred to nitrocellulose membranes. After blocking with 5% skimmed milk for 2 h, membranes were incubated with the following primary antibodies at 4 °C overnight: NLRP3 (Proteintech, Chicago, USA, 1:1000, Cat. No.: 19771-1-AP), caspase-1 (Proteintech, Chicago, USA, 1:1000, Cat. No.: 22915-1-AP), GSDMD (Santa Cruz, USA, 1:500, Cat. No.: A2315), IL-1β (ABclonal, Boston, USA, 1:1000, Cat. No.: A1112), IL-18 (ABclonal, Boston, USA, 1:1000, Cat. No.: A1115), Transferrin (BIOSS, Beijing, China, 1:1000, Cat. No: bs-2052R), or GAPDH (Proteintech, Chicago, USA, 1:2000, Cat. No: 60004-1-lg). After washing with TBST, membranes were incubated with HRP-conjugated secondary antibody (1:10,000) for 2 h. Western blot bands were analyzed with the ChemiDicTM XRS+ Imaging System (Bio-Rad Laboratories, Hercules, CA, USA), and the band densities were quantified with Multi Gauge Software of Science Lab 2006 (FUJIFILM Corporation, Tokyo, Japan).

### Immunofluorescence

Immunofluorescence staining for endothelial cells was performed using standard protocols. In Brief, the cells were fixed with 4% paraformaldehyde for 30 min, penetrated with 0.3% Triton X-100 for 1 h, and then blocked with 5% bovine albumin for 30 min. Subsequently, cells were incubated with anti-caspase-1 antibody (Proteintech, Chicago, USA, 1:50, Cat. No.: 22915-1-AP) at 4 °C overnight, followed by incubation at 37 °C with an Alexa Fluor 594 donkey anti-rabbit secondary antibody (Abcam, USA, 1:1000, Cat. No.: ab150076) for 1 h. Nuclei were stained by DAPI (Beyotime, China) for 20 min. Then the cells were photographed under a laser scanning confocal microscope (Nikon, A1 PLUS, Tokyo, Japan).

### DNA fragmentation evaluation

DNA fragmentation of endothelial cells was measured by TUNEL staining assay as previously described^13^. Briefly, endothelial cells were cultured on coverslips in a 24-well plate. After the indicated treatments, the cells were fixed with 4% paraformaldehyde and permeabilized with 0.3% Triton X-100. After washing with PBS, cells were incubated with TUNEL reaction mixture at 37 °C in the dark for 1 h, and stained by DAPI. Then cells were examined under a confocal laser scanning microscope (Nikon, A1 PLUS, Tokyo, Japan).

### Cell death assay

Pyroptotic cell death in endothelial cells was assessed using LDH release assay and Hoechst 33342/PI staining^11^. After treatment with sera for 24 h, cell culture medium was replaced with DMEM. After 6-h exposure, the supernatants from the treated cells were collected and centrifuged (400 × *g*, 5 min). Then 120 µl of the supernatant from each sample was transferred to a new 96-well plate and mixed with 60 µl reaction mixture (20 µl lactate, 20 µl INT, and 20 µl diaphorase) for 30 min at room temperature. Serum-free medium was used as the 0% control and lysates of the untreated cell were used as the 100% maximal release. The absorbance was measured at 450 nm on a spectrophotometric microplate reader. For Hoechst 33342/PI staining (Beyotime Institute of Biotechnology, Haimen, China), the treated cells were collected and resuspended in staining buffer, then incubated with a mixed solution of Hoechst 33342 and PI for 25 min at 4 °C. After that, the cells were photographed under an inverted fluorescence microscope (Nikon, TE-2000 U).

### Preparation of CAWS

The CAWS were prepared from *Candida albicans* strain NBRC1385 by previously described methods^5,40,45^. Briefly, *C. albicans* cultures were incubated in C-limiting medium at 27 °C for 2 days at a rotation speed of 270 rpm. After that, an equal volume of ethanol was added and put in a refrigerator at 4 °C overnight. Then the cultures were collected by centrifugation, and the pellet was dissolved in water with stirring for 2 h. Next, the complex was centrifuged again, and the soluble fraction was harvested and mixed with an equal volume of ethanol, and allowed to stand undisturbed overnight. Finally, the complex was centrifuged, and the precipitate was acquired to dry with acetone. The obtained CAWS were dissolved in 0.9% normal saline and autoclaved before use.

### Animals and ethics statements

For animal models of KD, male C57BL/6 mice (3–4 weeks of age) obtained from Wenzhou Medical University, License No. SCXK [ZJ] 2005-0019, were kept at a standard experiment cage in controlled conditions with temperature of 23 ± 2 °C, and humidity of 50 ± 5%. Mice were divided into six groups (*n* = 6 for each group) using a method of randomization: PBS group, CAWS group, CAWS + MCC950 group, CAWS + CA074-Me group, CAWS + FPS-ZM1 group, and CAWS + Anti-HMGB1 group. For groups designated for inhibitor pretreatment, inhibitors were injected 60 min before CAWS (4 mg/body) were intraperitoneally administered. These procedures were repeated for 4 days. At day 28 post-final CAWS injection, the mice were anesthetized and killed for harvest of heart tissues and follow-up examinations. All experiments were conducted according to the Guide for the Care and Use of Laboratory Animals of the China National Institutes of Health, and were authorized by the Animal Care and Use Committee of Wenzhou Medical University (wydw 2017-0046).

### Histology and immunostaining

Animal hearts were harvested and then fixed with 4% paraformaldehyde and embedded with paraffin. 5 μm-thick sections were obtained, and HE staining was performed using standard protocols. TUNEL staining and immunostaining of caspase-1 and CD31 (endothelial cell marker) on the coronary arteries were also done. Mouse monoclonal anti-CD31 antibody (ab24950) and an Alexa Fluor 594 donkey anti-mouse secondary antibody (ab150108) were purchased from Abcam (USA). Images were examined under a laser scanning confocal microscope (Nikon, A1 PLUS, Tokyo, Japan).

### Data analysis

Data were presented as mean ± SD, and were analyzed using SPSS version 17.0. All the data were normally distributed. A two-tailed unpaired Student’s *t*-test was conducted to compare two experimental groups, and one way analysis of variance (ANOVA) followed by Duncan’s multiple-range test was performed for the comparison of more than two groups. *P*-values less than 0.05 were considered as statistically significant. Sample size was chosen according to previous reports^2,5,7^, which performed similar experiments to observe significant results. Variance was similar between the groups that were being statistically compared.

## Supplementary information

Figure S1

Figure S2

Figure S3

Figure S4

Figure S5

Figure S6

Supplementary Figure Legends

Reproducibility Checklist

Detailed Attribution of Authorship
