# Supplementary figures and images for: Endothelial cell pyroptosis plays an important role in Kawasaki disease via HMGB1/RAGE/cathespin B signaling pathway and NLRP3 inflammasome activation
Source: Cell Death Dis. 2019 Oct 14;10(10):778. doi: 10.1038/s41419-019-2021-3 (PMC6791856; doi:10.1038/s41419-019-2021-3)

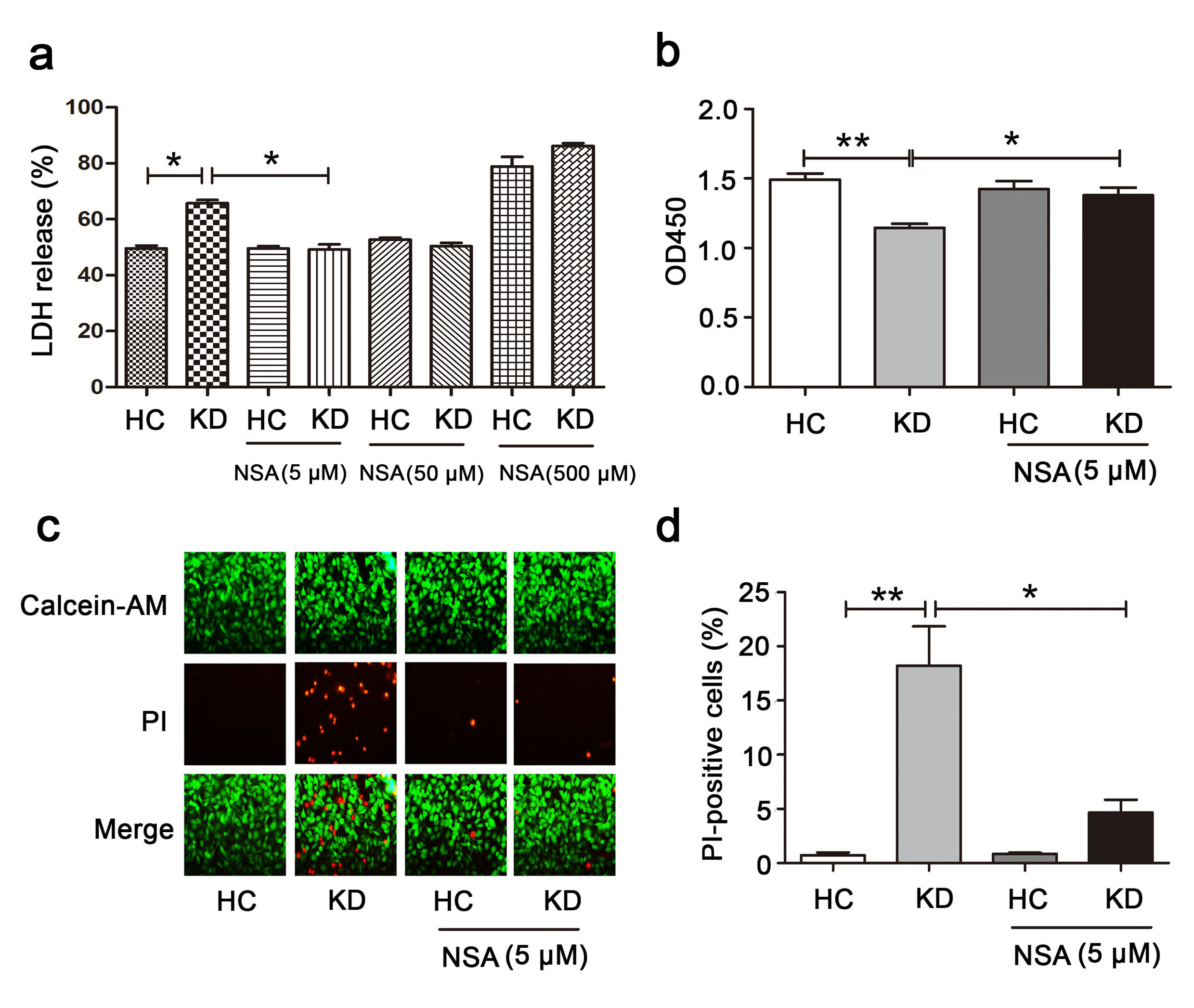

Supplement: Supplementary file 1 — Figure S1 [file 41419_2019_2021_MOESM1_ESM.tif]

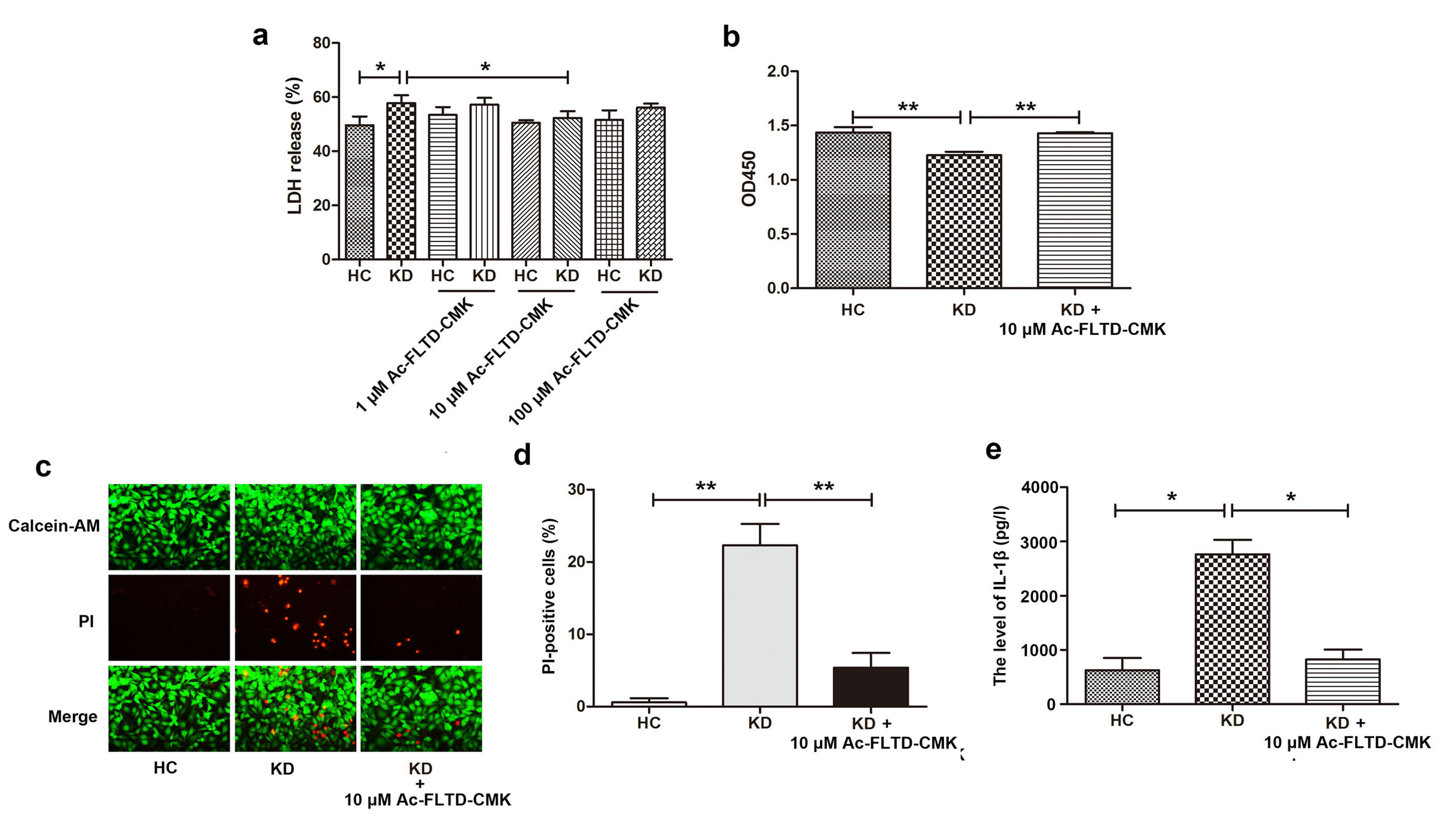

Supplement: Supplementary file 2 — Figure S2 [file 41419_2019_2021_MOESM2_ESM.tif]

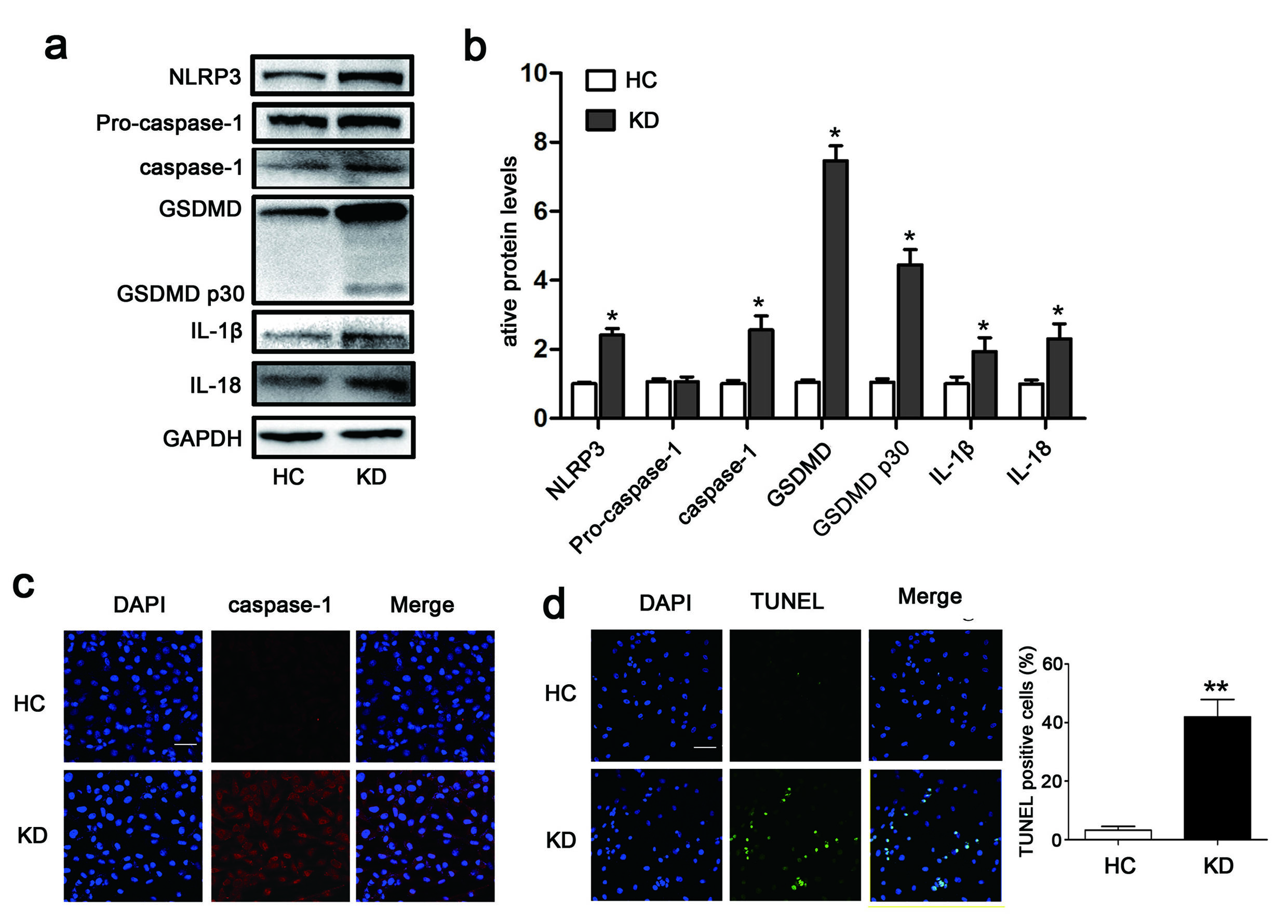

Supplement: Supplementary file 3 — Figure S3 [file 41419_2019_2021_MOESM3_ESM.tif]

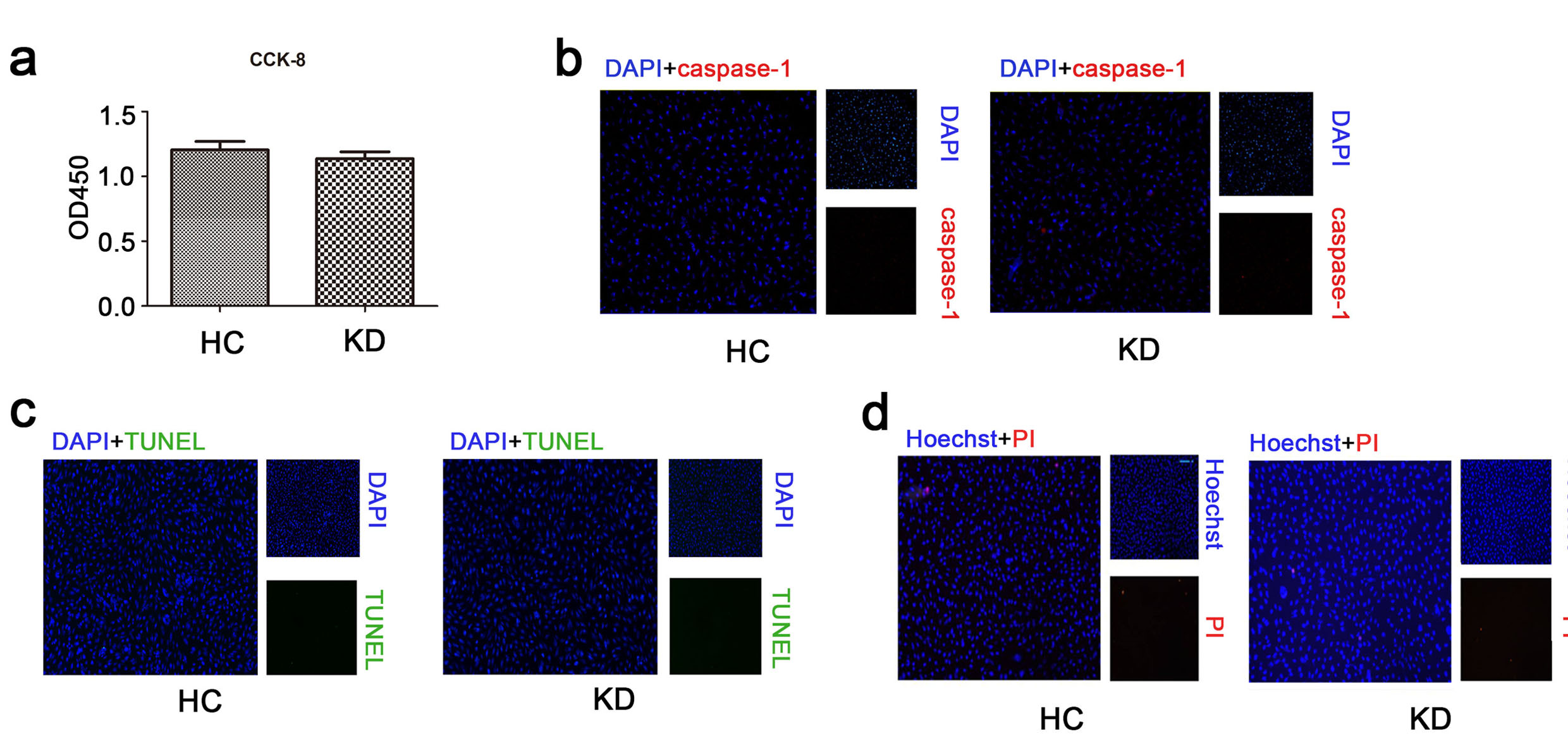

Supplement: Supplementary file 4 — Figure S4 [file 41419_2019_2021_MOESM4_ESM.tif]

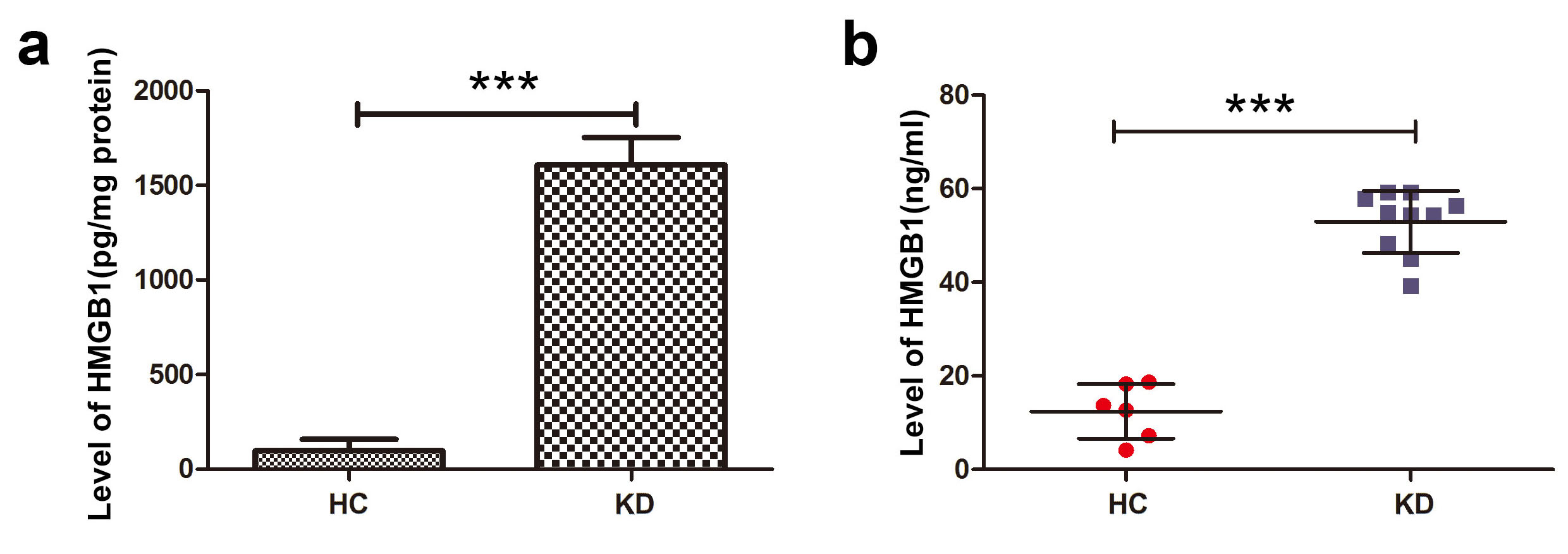

Supplement: Supplementary file 5 — Figure S5 [file 41419_2019_2021_MOESM5_ESM.tif]

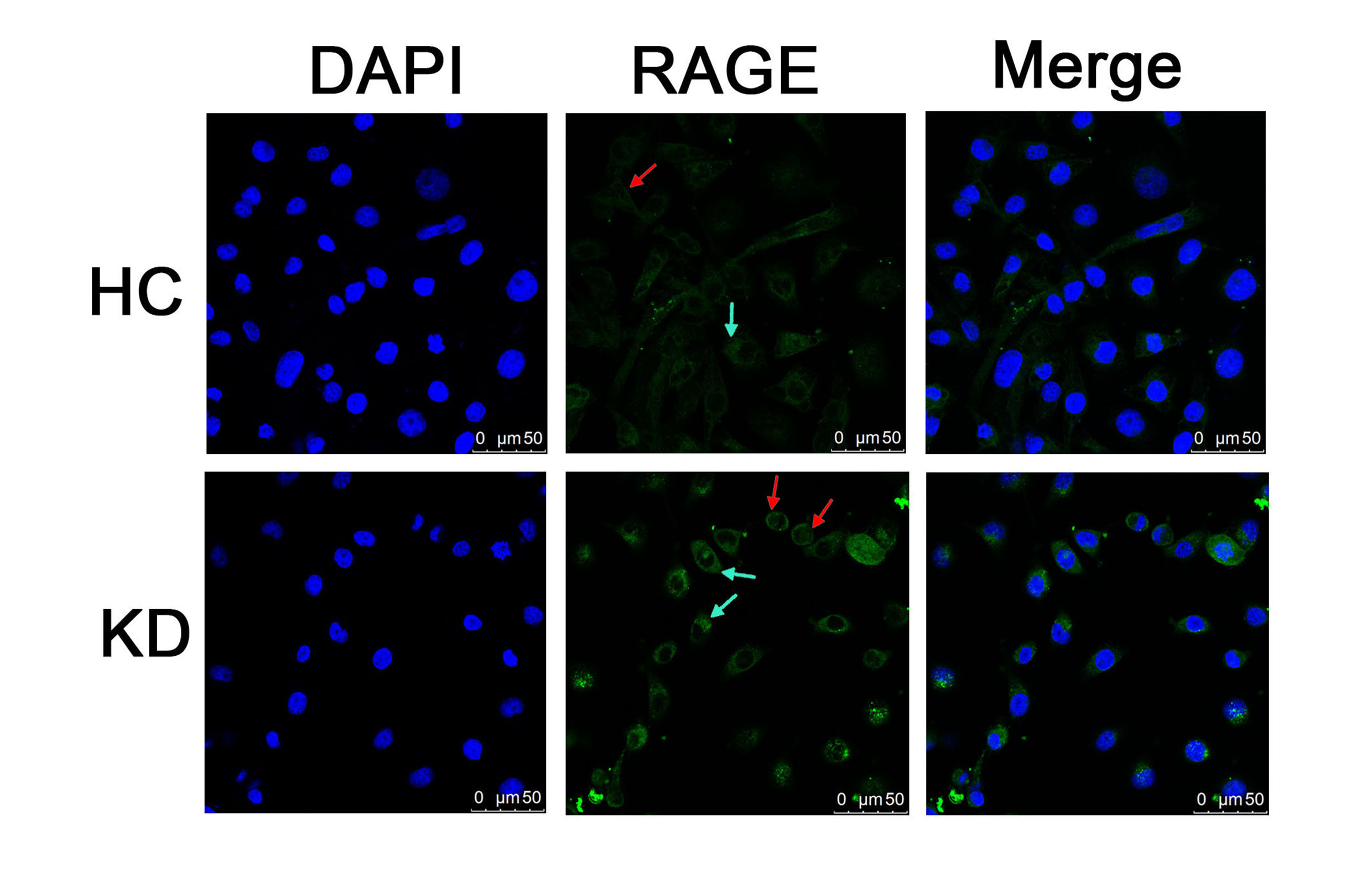

Supplement: Supplementary file 6 — Figure S6 [file 41419_2019_2021_MOESM6_ESM.tif]
