# Supplementary material for: Endothelial cell pyroptosis plays an important role in Kawasaki disease via HMGB1/RAGE/cathespin B signaling pathway and NLRP3 inflammasome activation
Source: Cell Death Dis. 2019 Oct 14;10(10):778. doi: 10.1038/s41419-019-2021-3 (PMC6791856; doi:10.1038/s41419-019-2021-3)
Supplement: Supplementary file 7 — Supplementary Figure Legends [file 41419_2019_2021_MOESM7_ESM.docx]

**Fig. S1 Effect of the pyroptosis inhibitor, necrosulfonamide (NSA), on LDH release (a), viability (b), survival (c), and the percentage of PI-positive cells (d) in KD-treated ECs.*** *P*<0.05, and ** *P*<0.01 indicated significant difference between KD- and HC-treated ECs, or KD-treated ECs with and without NSA addition.

**Fig. S2 Effect of the GSDMD-derived inhibitor, *N*-acetyl-Phe-Leu-Thr-Asp- chloromethylketone (Ac-FLTD-CMK), on LDH release (a), viability (b), survival (c), the percentage of PI-positive cells (d), and IL-1β release (e) in KD-treated ECs.** * *P*<0.05, ** *P*<0.01 indicated significant difference between KD- and HC-treated ECs, or KD-treated ECs with and without Ac-FLTD-CMK pretreatment.

**Fig. S3 Pyroptosis-related parameters were upregulated in human umbilical vein endothelial cells (HUVECs) after exposure to KD sera-treated PBMCs.** **(a-b)** Protein levels of caspase-1, GSDMD, cleaved p30 form of GSDMD, mature IL-1β and IL-18 were examined by western blot analysis. GAPDH was used as an internal control. Data were shown as mean ± SD (n=3). * P<0.05. **(c-d)** Caspase-1 fluorescence intensity and the percentage of TUNEL-positive cells were respectively evaluated using immunofluorescent assay and TUNEL staining. The nuclei were stained blue using DAPI. Magnification: ×200. Scale bar=100 μm. ***P* < 0.01.

**Fig. S4 Effect of KD sera on EC viability (a), caspase-1 expression (b), DNA fragmentation (c), and cell membrane rupture (d).**

**Fig. S5 HMGB1 levels were determined in HC/KD sera-treated THP1 cells (a), and HC/KD sera (b).** ****P*<0.001 indicated significant difference between KD sera-treated and HC sera-treated THP1 cells, or between KD sera and HC sera.

**Fig. S6 The cellular localization of RAGE was examined in KD-treated ECs.** Red arrows indicated the plasma membrane localization of RAGE, and the blue arrows pointed to the cytoplasmic distribution of RAGE.
