# Supplementary material for: Endothelial cell pyroptosis plays an important role in Kawasaki disease via HMGB1/RAGE/cathespin B signaling pathway and NLRP3 inflammasome activation
Source: Cell Death Dis. 2019 Oct 14;10(10):778. doi: 10.1038/s41419-019-2021-3 (PMC6791856; doi:10.1038/s41419-019-2021-3)
Supplement: Supplementary file 9 — Detailed Attribution of Authorship [file 41419_2019_2021_MOESM9_ESM.pdf]

DECLARATION OF CONTRIBUTIONS TO ARTICLE

ADMC

Manuscript Number: CDDIS-19-1868R

Journal Name: ~~*Cell Death & Differentiation*~~ (the 'Journal')

Proposed Title of the Contribution:

Endothelial cell pyroptosis plays an important role in Kawasaki Disease via HMGB1/RAGE/ Cathespin B signaling pathway and NLRP3 inflammasome activation (the 'Contribution)

Author(s):

Chang Jia, Jian Zhang, Huanwen Chen, Yingzhi Zhuge, Huiqiao Chen, Fanyu Qian, Kailiang Zhou, Chao Niu, Fangyan Wang, Huixian Qiu, Zhenquan Wang, Jian Xiao, Xing Rong, Maoping Chu (the 'Authors')

For all *CDD* articles, each person named as an author in the published version must be able to show he or she has contributed substantially to the article.

Authorship credit should be based on 1) substantial contributions to conception and design, acquisition of data, or analysis and interpretation of data; 2) drafting the article or revising it critically for important intellectual content; and 3) final approval of the version to be published. Authors should meet conditions 1, 2 and 3.

Any person who cannot be shown to have made a substantial contribution to the article cannot be listed as an author in the final version. The name of any person who is deemed to have made a minor contribution can, however, appear in the Acknowledgments section of the article.

Please complete the table below to indicate the contributions of all named authors to the manuscript.

Author Full Name:	Specification of Contribution to the Manuscript:
Chang Jia	design and acquire substantial data; analyze and interpret data; draft and revise the manuscript; final approval of the version to be published
Jian Zhang	acquire substantial data; supplement experiments and revise the manuscript; final approval of the version to be published
Huanwen Chen	analyze and interpret data; draft and modify the manuscript critically; final approval of the version to be published
Yingzhi Zhuge	acquire some data; revise the manuscript for important intellectual content; final approval of the version to be published
Huiqiao Chen	acquire some data; revise the manuscript; final approval of the version to be published
Fanyu Qian	acquire some data; revise the manuscript; final approval of the version to be published
Kailiang Zhou	acquire some data; revise the manuscript; final approval of the version to be published
Chao Niu	acquire some data; revise the manuscript; final approval of the version to be published
Fangyan Wang	acquire some data; revise the manuscript; final approval of the version to be published
Huixian Qiu	collect and analyze the clinical specimens; revise the manuscript; final approval of the version to be published
Zhenquan Wang	collect and analyze the clinical specimens; revise the manuscript; final approval of the version to be published
Jian Xiao	analyze and interpret data;modify the manuscript critically; final approval of the version to be published
Xing Rong	design the study; revise the manuscript substantially; final approval of the version to be published

Maoping Chu

Figure 7:
Jian Zhang, Kailiang zhou and Chao Niu maked the KD animal model;
Kailiang Zhou, Chao Niu, Xing Rong and Fangyan Wang was responsible for HE and IHC staining, and determination of the fluorescence intensity of caspase-1 and TUNEL in animal heart tissue.
Jian Zhang, Huiqiao Chen and Fangyan Wang determined the expression of pyrotposis-related proteins by western blot analysis;
Jiao Xiao analyzed and interpreted the data.

Figure 8:
Chang Jia, Xing Rong and Maoping Chu drew the schematic model for endothelial cell pyroptosis in Kawasaki disease.

Figure 1:

Zhenquan Wang and Huixian Qiu collected the sera from HC and KD patients;
Chang Jia, Jian Zhang and Yingzhi Zhuge determined the levels of these proteins by ELISA;
Jian Zhang examined the protein expression of GSDMD and IL-1β by western blot analysis;
Zhenquan Wang and Huixian Qiu analyzed the above data.

Figure 2:

Zhenquan Wang and Huixian Qiu collected the sera from HC and KD patients;
Jian Zhang examined the expression of pyroptosis-related proteins;
Yingzhi Zhuge determined the fluorescence intensity of caspase-1 and TUNEL;
Fanyu Qian examined LDH release and conducted Hoechst 33342/PI staining;
Huanwen Chen analyzed and interpret the data.

Figure 3:

Zhenquan Wang and Huixian Qiu collected the sera from HC and KD patients;
Jian Zhang and Huiqiao Chen examined the expression of pyroptosis-related proteins;
Chang Jia determined the fluorescence intensity of caspase-1andTUNEL, LDH release and conducted Hoechst 33342/PI staining;
Chang Jia analyzed and interpret the data.

Figure 4:

Zhenquan Wang and Huixian Qiu collected the sera from HC and KD patients;
Yingzhi Zhuge determined the activity of Cathepsin B and determined the fluorescence intensity of caspase-1an d TUNEL;
Jian Zhang and Huiqiao Chen examined the expression of pyroptosis-related proteins;
Fanyu Qian examined LDH release and conducted Hoechst 33342/PI staining;
Chang Jia and Huanwen Chen analyzed the data.

Figure 5:

Zhenquan Wang and Huixian Qiu collected the sera from HC and KD patients, and analyzed the HMGB1 contents in THP1 cells and medium supernatant;
Yingzhi Zhuge examined the mRNA and protein level of RAGE and determined the activity of Cathepsin B;
Jian Zhang and Huiqiao Chen examined the expression of pyroptosis-related proteins;determined the fluorescence intensity of caspase-1 and TUNEL.
Fanyu Qian examined LDH release and conducted Hoechst 33342/PI staining;
Chang Jia and Huanwen Chen analyzed the data.

Figure 6:

Jian Zhang, Yingzhi Zhuge, Huiqiao Chen and Fanyu Qian maked the KD animal model;
Kailiang Zhou and Chao Niu were responsible for HE and IHC staining;
Jian Zhang and Huiqiao Chen determined the expression of pyrotposis-related proteins by western blot analysis;
Xing Rong and Fangyan Wang examined the fluorescence intensity of caspase-1 and TUNEL in animal heart tissue.
Chang Jia and Huanwen Chen analyzed and interpreted the data.

Signed for and on behalf of the Author(s):	Print Name:	Date:
Maoping Chu	Maoping Chu	2019-9-19
